# Preliminary Assessment of Occurrence, Potential Origin, and Human Health Risk of Volatile Organic Compounds in Uncontrolled Springs, North Morocco

**DOI:** 10.3390/metabo12121213

**Published:** 2022-12-02

**Authors:** Wafae Lechhab, Fabrizio Cincotta, Touria Lechhab, Concetta Condurso, Farida Salmoun, Francesco Cacciola, Antonella Verzera

**Affiliations:** 1 Laboratory of Physical Chemistry of Materials, Natural Substances and Environment, Chemistry Department, Faculty of Sciences and Technology Tangier, Abdelmalek Essaâdi University, Tangier 90090, Morocco; 2 Department of Veterinary Sciences, University of Messina, Polo Universitario Dell’Annunziata, Viale G. Palatucci, 98168 Messina, Italy; 3 Department of Biomedical, Dental, Morphological and Functional Imaging Sciences, University of Messina, 98125 Messina, Italy

**Keywords:** springwater, volatile organic compounds, origin of pollution, health risk assessment, HS-SPME-GC/MS, North Morocco

## Abstract

In recent years, with the drastic increase in worldwide pollution rates, considerable attention has been paid to the volatile organic compounds (VOCs) that might lead to serious health problems, e.g., cancer. As there appears to be a notable lack of research on the pollution (specifically, VOCs) of water bodies in Morocco, we aimed to assess the occurrence of VOCs in some uncontrolled springs in the north of Morocco that have not been previously investigated. We also discuss the estimation of health risks posed by ingestion and dermal contact as well as the different potential origins of these pollutants. For this purpose, water samples were collected from twenty-six sampling sites and were analyzed via headspace solid-phase microextraction coupled with gas chromatography–mass spectrometry (HS-SPME-GC-MS). Out of the 60 suspected VOCs, a total of 15 compounds belonging to five distinct groups were identified and quantified. Among them, fumigants, solvents, and gasoline hydrocarbons were the most abundant groups, with proportions of 40%, 26.7%, and 20%, respectively. A heatmap clustered the provinces based on their degree of pollution, while a dendrogram was used to classify the studied springs into six main groups. Regarding carcinogenic risk, all the samples were safe for consumption as well as for dermal contact, except for S17, S18, and S8, and S19, which might present a severe threat to inhabitants due to their contents of, respectively, naphthalene (2.1 × 10^−3^), chloroform (2.5 × 10^−4^), and cis and trans-dichlropropene (1.61 × 10^−4^ and 1.11 × 10^−4^). Our investigation revealed several anthropogenic sources of water contamination, which could aid authorities in limiting contamination spread in water bodies.

## 1. Introduction

Water is not only the cradle of life, the indispensable staple substance for all life forms, but it is also a core pillar of economic growth. It is an essential element for good health that must be clean or at least palatable for human consumption. According to the United Nations of International Children’s Emergency Fund (UNICEF) and the World Health Organization (WHO), an estimated 435 million people worldwide still used unimproved sources of drinking water in 2017, including unprotected wells and springs. In Morocco, between 2000 and 2017, the proportion of the rural population using improved water supply without contamination increased by 34%, reaching 65% [1]. Despite this, due to the free access, embedded traditions, and the belief in water’s curative power, uncontrolled sources are still being used (particularly in the northern region) as the main source of drinking as well as hydration for agriculture and livestock. Unexpectedly, the majority of the urban population prefers to consume natural spring water more than any other kind, i.e., tap or bottled water.

As uncontrolled spring water contains dangerous substances, it mostly constitutes a threat to public health when it is the main source of the public water supply. Indeed, volatile organic compounds (VOCs) are among the contaminants of concern that are now widely released into the environment and migrate to drinking water supplies via the atmosphere, soil, surface water, and groundwater [2,3]. According to many scientific organizations and official agencies in different countries, VOCs consist of a wide range of organic compounds that have a relatively high vapor pressure with an initial boiling point less than or equal to 250 °C measured at a standard atmospheric pressure of 101.3 kP [4,5]. Regarding their origin, anthropogenic sources are numerous and include solvents, herbicides, pesticides, fumigants, paints, adhesives, deodorants, refrigerants, and gasoline or oil spills, but they are also naturally released by plants, animals, and microbes during the processes of growth, maintenance, and decomposition [6,7]. According to their predominant use or origin, VOCs are classified into fumigants, gasoline hydrocarbons, gasoline oxygenates, organic synthesis compounds, refrigerants, solvents, or trihalomethanes [8].

It is widely known that exposure to some VOCs may cause certain types of cancer [9], birth defects [10], leukemia [11,12], or damage to the immune system [13], central nervous system [14], liver, or kidneys [15]. Hence, the United States Environmental Protection Agency (EPA) has created several models to assess lifetime hazards for individuals that may be exposed to carcinogenic or non-carcinogenic risks due to chemicals from different environmental media such as surface water, groundwater, and drinking water [16]. To guarantee the safety of drinking water supplied to consumers, risk assessment provides support to planning and quality monitoring. Findings from such risk assessments can inform authority decision-making on use-restriction or the introduction of required changes [17].

Headspace solid-phase microextraction gas chromatography–mass spectrometry (HS-SPME-GCMS) is a reliable and beneficial method to assess the presence of VOCs in water samples [18,19,20]. It has successfully been applied to the analysis of trace levels of methyl tert-butyl ether (MTBE) in various water samples [21] and showed great analytical performance for chlorinated benzenes in natural water analysis [22]. Moreover, the extraction technique (headspace SPME) is used to extract a wide range of organic compounds from various matrices and is characterized by its simplicity, speed, accuracy, and environmental friendliness [18,23,24].

Spring water is a vital water source for ensuring rural water security in Morocco, especially in the northern region where springs are abundant. Insofar as this is the case, uncontrolled water sources (wells and springs) may pose the principal contamination risk for local inhabitants. Some previous studies have investigated heavy metals, physicochemical parameters, and biological indicators of pollution [25,26]; however, a deficiency of data was observed for VOCs.

This study was designed based on the following objectives: first, to investigate the occurrence of VOCs in twenty-six uncontrolled spring waters located in the northwestern area of Morocco and to fill the literature gap on VOCs in Moroccan spring waters; second, to identify the potential origins of pollution by underlining the main human activities causing the distribution of these pollutants in the environment; third, to assess the potential health risk of the detected VOCs through two main routes of exposure—ingestion and dermal.

By evaluating health risks and gathering evidence on pollution sources, this first investigation into VOCs in Moroccan spring waters will be useful for environmental researchers and local authorities, whose water resource protection policies can significantly contribute to guaranteeing the safety of drinking water. This study seeks to pave the way for future research on water pollution and human health by supporting the global goal of “safe water for all”.

## 2. Materials and Methods

### 2.1. Study Area

**Figure 1:**
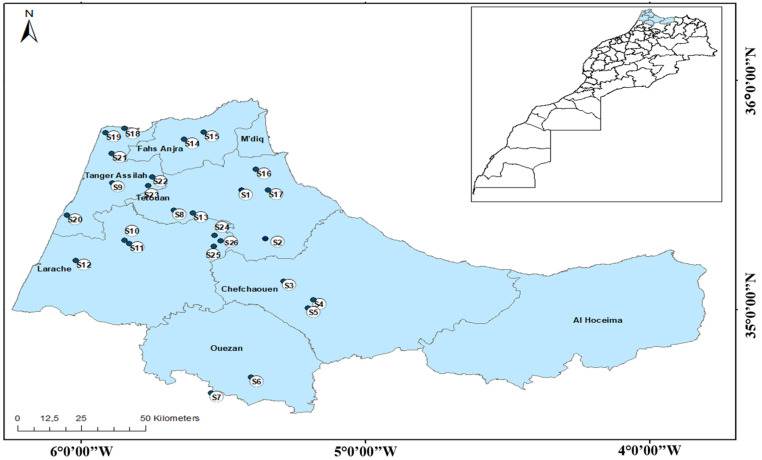
Study area and sampling locations of the studied spring waters in northern Morocco.

Water samples were collected from several points (Figure 1) located in the northwestern area of Morocco (Tangier–Tetouan–Al Hoceima region).According to the last census in 2014, the region has six provinces (Chefchaouen, Larache, Tetouan, Al Hoceima, Ouazzane, and Fahs-Anjra) and two prefectures (Tangier-Asilah and M’Diq-Fnideq) with a total mid-year population of approximately 3,813,854 [27]. The wet season is October-March and the annual average rainfall varies from about 400 up to over 1000 mm according to the geographical position [28]. More precisely, along the entire western coast between Larache and Martil, the rainfall can exceed 700 mm/year, while in the eastern part, it reaches 400 mm/year. At high reliefs, the average rainfall varies between 600 and 1800 mm [29]. All information on the studied spring waters regarding location, province, population and households, households connected to public water supply, wastewater disposal methods, methods of domestic waste disposal, type of spring, and consumers is reported in Table S1 and derived from the High Commission for Planning of Morocco [30].

### 2.2. Sampling

In November 2020, water samples from twenty-six uncontrolled Moroccan springs were collected in duplicate. To avoid the possible loss of volatile analytes, the sampling was conducted according to the conditions established by EPA method 524.2. In detail, water was filled directly from the source in 60 mL glass vials with Teflon^®^ septa. Once the cap was tight, the vials were inverted to check for the presence of air bubbles, and then stored immediately at 4 °C. All samples were analyzed within 7 days of sampling, with two replicates each.

### 2.3. Chemicals and Reagents

Volatiles Organic Mix EPA 502/524 containing 60 VOCs at a concentration of 200 µg/mL-methanol for each compound was purchased from Sigma-Aldrich. Heptane, which was used as the internal standard (IS), acetone, and methanol were obtained from Merck, Milan with purities of 99%, 99.5%, and 99.8%, respectively. Sodium chloride (NaCl) of analytical grade was purchased from J&K Scientific Limit (Beijing, China), and ultrapure water was obtained from a Milli-Q purification system (Millipore, Bedford, MA, USA).

### 2.4. VOC Extraction

**Figure 2:**
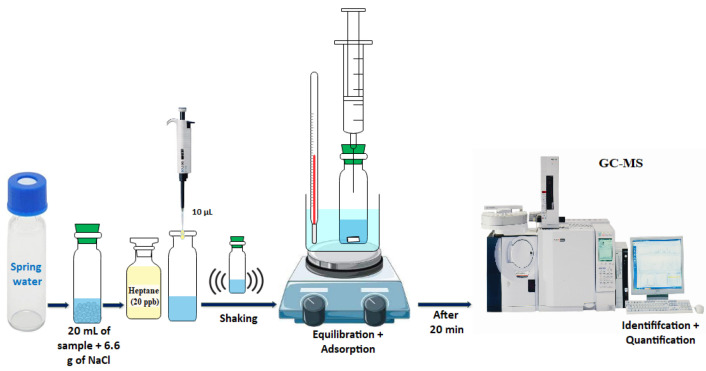
VOC extraction from North Moroccan spring water samples.

For the extraction of VOCs, the headspace solid-phase microextraction (HS-SPME) technique was used (Figure 2). A carboxen/polydimethylsiloxane (carboxen/PDMS) fiber of 85 μm film thickness (Agilent, Milan, Italy) housed in its manual holder (Supelco, Bellefonte, PA, USA) was used, and it was conditioned according to the manufacturer’s instructions before use.

Briefly, 20 mL of water sample and 6.6 g of NaCl were added in a 40 mL vial equipped with a “mininert” valve (Supelco, Bellefonte, PA, USA). Afterwards, the sample was equilibrated for 10 min and then extracted for 10 min at 35 °C under constant stirring. After the extraction, the SPME fiber was injected at 260 °C in the GC/MS injector in splitless mode and was held for 3 min for the desorption of the analytes. After each extraction, the fiber was cleaned at operating temperature and its purity was checked.

#### Gas Chromatography–Mass Spectrometry (GC-MS) Analysis

A Shimadzu GC 2010 Plus gas chromatograph directly interfaced with a TQMS 8040 triple quadrupole mass spectrometer (Shimadzu, Milan, Italy) operating in selected ion monitoring (SIM) was used for the analysis. Chromatographic conditions were as follows: Column DB-624, 25 m × 0.200 mm i.d. × 1.12 μm (Agilent US0445022H); injection temperature 260 °C, splitless mode, oven temperature 40 °C held for 5 min, 40 °C to 180 °C at 8 °C/min, 180 to 260 °C at 30 °C/min, 260 °C held for 1 min; helium as carrier gas with a constant column flow of 0.8 mL/min, purge flow 1 mL/min; transfer line temperature, 250 °C. Mass spectra were recorded with Mode SIM Scan at an event time of 0.03 s/scan.

The identification of the compounds was conducted using mass spectral data from the NIST’18 (NIST/EPA/NIH Mass Spectra Library, version 2.0, Gaithersburg, MD, USA) database, as well as the injected standards (EPA 502 /524.2 VOCs Mixture).

### 2.5. Quantitative Analysis

The quantitation of VOCs was based on a five-point calibration curve generated by plotting detector response versus the amount spiked from each external standard. The mother solution (EPA standard) was diluted in methanol to prepare the desired standard concentrations. Then, similar to the samples, the different standard concentrations were extracted and analyzed by HS-SPME-GC-MS. The limits of quantification (MQL) and detection (MDL) of the method were calculated from specific calibration curves constructed using blank samples containing the analytes in the range of their limits of detection. The slope (m) and the standard deviation of y-intercept (σ) of the regression lines were thus used for MDL and MQL calculation using Equations (1) and (2), respectively.

MDL = 3 × σ/m(1)

MQL = 10 × σ/m(2)

where σ is the standard deviation of the intercept and m is the slope of the calibration curve. To ensure the quality of the assay control, at the same time as the samples, 10 µL of heptane (20 ppb) was spiked as an internal standard. Table S2 shows the data for qualitative ion, quantitative ion, R^2^_,_ retention time, MDL, and MQL of 15 detected VOCs.

### 2.6. Statistical Analysis

The results of GC-MS analysis are expressed in µg/L as the mean value of the detected VOCs and standard deviation of four measurements (mean ± SD). ArcGIS desktop 10.6.1 software was used to visualize the location of sampling sites, and the R software (version 4.1.2) package was used to perform the heatmap and the hierarchical cluster analysis of principal components (HCPC).

### 2.7. Health Risk Assessment

#### 2.7.1. Carcinogenic Risk

For carcinogenic effects, the health risk was estimated using Formula (3):

(3) Risk= CDI ×SF when Risk<0.01 

However, for the chief pathway (ingestion), the formula of chronic daily intake (CDI), also known as lifetime average daily dose (LADD), was applied to calculate the cancer risk (Equation 4) [31].

(4) $$\;\mathrm{CDI}\;\left(\frac{\mathrm{mg}}{\mathrm{Kg}}-\mathrm{day}\right)=\frac{\;\mathrm{Cw}\;\times \;\mathrm{IR}\;\times \;\mathrm{EF}\;\times \;\mathrm{ED}\;}{\mathrm{BW}\;\times \;\mathrm{AT}}$$

where SF is the slope factor, expressed in (mg/kg-day)^−1^, CDI is the chronic daily intake averaged over 70 years (mg/kg-day)^−1^, Cw is the concentration in water (mg/litter), IR is the ingestion rate (liter/day), EF is the exposure frequency (days/year), ED is the exposure duration (years), BW is the body weight (Kg), and AT is the averaging time (days).

For dermal absorption routes, Equation (5) [32]—

(5) Dermal cancer risk=DAD×SFABS

—was used with Equations (6) and (7)—

(6) $$\mathrm{SFABS}=\frac{\mathrm{SFo}}{\mathrm{ABSGI}}$$

and

(7) $$\mathrm{DAD}\;\left(\frac{\mathrm{mg}}{\mathrm{Kg}}-\mathrm{day}\right)=\frac{{\mathrm{DA}}_{\mathrm{event}}\times \;\mathrm{EV}\;\times \;\mathrm{ED}\;\times \;\mathrm{EF}\;\times \;\mathrm{SA}}{\mathrm{BW}\;\times \;\mathrm{AT}}\;\;\;\;$$

—where $\;{\mathrm{DA}}_{\mathrm{event}}$ (mg/cm^2^-event) is calculated for organic compounds as follows:

If t event ≤ t*, then: ${\mathrm{DA}}_{\mathrm{event}}=\frac{2\;\mathrm{FA}\;\times \;\mathrm{Kp}\;\times \;\mathrm{Cw}\;\surd \left(6\tau \;\mathrm{event}\;\times \;t\;\mathrm{event}\right)}{\;\pi }$.

Here, DAD is the dermal absorbed dose (mg/kg-day), SF_ABS_ is the absorbed cancer slope factor (mg/kg-day)^−1^, SF_O_ is the oral slope factor (mg/kg-day)**^−^**^1^, ABS_GI_ is the fraction of contaminant absorbed in the gastrointestinal tract in the critical toxicity study (dimensionless), ${\mathrm{DA}}_{\mathrm{event}}$ is the absorbed dose per event (mg/cm^2^-event), SA is the skin surface area available for contact (cm^2^), EV is the event frequency (events/day), EF is the exposure frequency (days/year), ED is the exposure duration (years), BW is the body weight (Kg), AT is the averaging time (days), FA is the fraction absorbed (dimensionless), Kp is the dermal permeability coefficient of compound (cm/h), Cw is the chemical concentration in water (mg/cm^3^), τ event is the lag time per event (h/event), t event is the event duration (h/event), and t* is the time to reach steady-state (h) = 2.4 τevent.

Table S3 exhibits the parameters and input assumptions for exposure assessment of volatile organic compounds through ingestion and dermal pathways [32,33].

#### 2.7.2. Non-Carcinogenic Risk

The chronic non-cancer risk from ingestion and dermal pathways was assessed with the risk quotient (HQ) method. The hazard quotient (HQ) through the principal route is equal to CDI/RfDo. If HQ = <1: no hazard exists; if HQ > 1: there is a possibility of non-carcinogenic risk.

**Figure 3:**
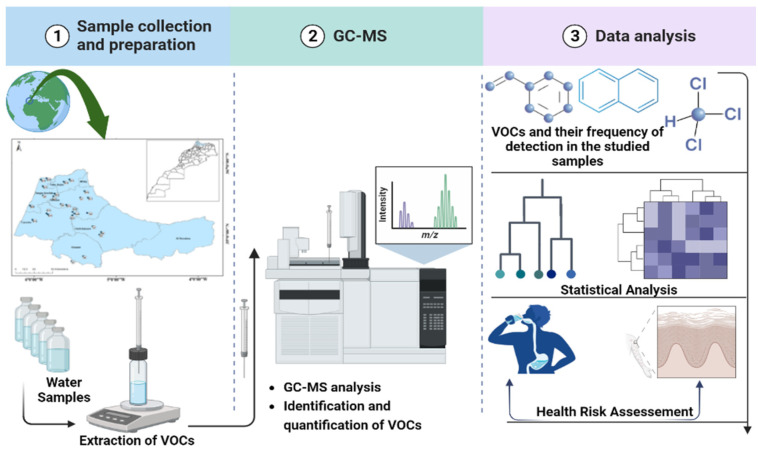
Flowchart of the experimental design employed in the present work.

The quotient of dermal non-cancer Risk is calculated as follows: DAD/RfD_ABS_, where RfD_ABS_ = RfD_O_ × ABS_GI_, and RfD_ABS_ is the absorbed reference dose (mg/kg-day) and RfD_O_ is the oral reference dose (mg/kg-day). Table S4 presents the slope factor, oral reference dose, and dermal permeability coefficient of the 15 detected VOCs according to the USEPA database [34]. A flowchart of the experimental design used in this study is provided in Figure 3.

## 3. Results and Discussion

### 3.1. General Characteristics

As the population and economic growth increase the demand for safe drinking water, assessing water quality by investigating the occurrence of contaminants in water bodies is crucial [8]. The assessment of VOCs in North Moroccan uncontrolled spring waters was carried out via HS-SPME-GC-MS, and the results were expressed in ppm. Table 1 reports the quantitative data of the detected VOCs in the twenty-six studied samples.

Out of 60, a total of 15 compounds belonging to five distinct groups (fumigant, solvent, gasoline hydrocarbon, organic synthesis compound, and trihalomethane) were identified and quantified. Following Zogorski, these volatile groups were classified according to their primary use [3]. Within the context, accounting for 40%, fumigant (2,2-DCP, 1,2-DCP, DBE, Cis-1,3-DCP, Trans-1,3-DCP, and 1,3-DCP) was the most prevalent class, followed by solvents (PCE, Trans-1,2-DCE, 1,3,5-TMB, and CYM) with 26.7%, gasoline hydrocarbon (NAPH, STY, and 1,2,4-TMB) with 20%, and finally organic synthesis compound (1,1-DCE) and trihalomethane (CHF) with an average of 6.7%, equally. Likewise, Rowe et al. [8] found seven distinct VOCs groups in non-treated well samples, and the predominant groups were as follows: solvent (36.85%), gasoline hydrocarbon (24.39%), fumigant (9.75%), and trihalomethane (9.75%). On the other hand, the lowest detected VOC in the investigated samples was Cis-1,3-DCP with 0.19 µg/L in S8. The highest detected VOC was CHF with 7.6 μg/L of in S18, followed by STY and PCE with 4.09 µg/L and 2.86 µg/L, respectively. Notably, all the detected chemicals in the investigated samples were below the established range by the WHO for drinking water [35]. However, in contradiction with earlier findings, much higher values were found for chloroform compared with those reported by Ikem [36]. Ikem found 0.1–0.3 µg/L in spring waters, in accordance with other values reported in the literature, e.g., [37,38] for surface waters in Greece (<MDL–1.5 µg/L) and China (0.012–1.4 µg/L), respectively. Insofar as only two of the 26 studied springs showed the presence of chloroform, and no other trihalomethane component associated with chlorination was detected in the investigated water samples, the findings reported in this work fit, to a certain extent, those reported by Page et al. [39], in which only 1 of the 147 studied springs was contaminated by chloroform, at 3.7 µg/kg.

Since the result of styrene was not previously discussed for springs, but only for some bottled spring waters [40], the findings reported in this work might be compared with those reported by Yu et al. [41], where the quantity of styrene varied between 0.1 and 6.5 µg/L in Korean underground water. In Morocco, there is a significant lack of research on VOCs in water bodies except for a study carried out by Amezghal et al. [42], who investigated VOCs in a dam in the Fez region. However, despite the authors’ claim that styrene was at high enough concentration for the odor could be detected, no quantitative information was provided.

PCE (tetrachloroethylene) exhibited the third-highest concentration (2.86 µg/L in S17) of the investigated samples. In contrast, concentrations of this volatile were found to range from <MDL to 8210 µg/L in different tap water sources around Wuhan’s river in China [43]. In fact, such high concentrations are usually revealed only in waters from industrial zones, such as in one study that revealed a range of 0.1 to 6000 µg/L in five Korean industrial complexes [41]. Notably, the main origins of tetrachloroethylene are industrial and agricultural activities that involve populated areas [19,37];dry-cleaning, vapor degreasing in metal-cleaning operations [44], and traffic-related sources [45]. However, to the best of our knowledge, no previous research has detected tetrachloroethylene in spring waters. Therefore, although the results reported in the present work differ considerably from those reported in refs. [19,37,46] the findings of this work cannot be directly compared to those from research conducted on different kinds of water sources (groundwater and surface water such as lakes and rivers).

As the detection of different amounts of VOCs in each country is likely to be correlated with point sources or diffuse pollution near the sampling sites depending on that country’s use of specific chemical compounds, differing geographical origins make our results incomparable to those of previous studies.

Of the samples that we investigated, 73% revealed the existence of some of our examined VOCs: about 38.46% contained VOC mixtures and 34.62% had a single VOC detection. To some extent, these findings fit those of Rowe et al. [8], who detected VOCs in 65% (31% and 34% with a single or a mixture of VOC(s), respectively) of their samples, but differ from Liu et al., where at least one VOC in the studied sampling sites was found [46].

**Figure 4:**
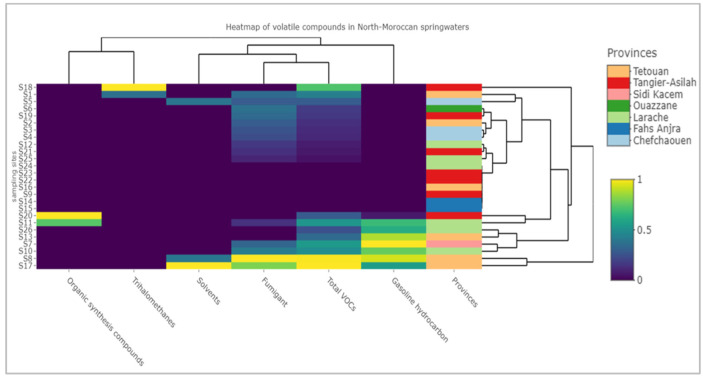
Heatmap of the volatile groups and total volatiles detected in twenty-six Moroccan spring waters based on the province of origin. Data were normalized, and the plotly method was used.

To better understand the distribution of the mixture of VOCs in the studied area, a heatmap was constructed to highlight the distribution and the abundance of compound classes at the sampling sites based on their province of origin. It is apparent from the heatmap (Figure 4) that VOCs were distributed throughout most of the sampled springs; however, seven sampling sites (S9, S14, S15, S16, S22, S23, and S24) were not contaminated by any VOC. In contrast, S17, S8, and S18 were the springs with the highest amount of total volatiles.

Generally, the clusters in the bottom regroup the most polluted sites by mixtures of volatile groups. These sampling areas belong mainly to Tetouan, Larache, and Sidi Kacem provinces and were mostly contaminated by fumigants, gasoline hydrocarbons, solvents, and organic synthesis compounds. While the other springs were not well-classified in some cases, depending on their geographical origins, most of theis samples (excluding the safe springs) contained fumigants. Notably, all springs in Fahs-Anjra province were VOC-free. Similarly, half of the Tangier-Asilah springs were also VOC-free, but the remainder contained one volatile group each (S19, S20: fumigant, S18: trihalomethanes, and S20: organic synthesis compounds). Spring waters from Chefchaouen province were mainly polluted by fumigants and solvents.

In sum, the heatmap successfully classified VOCs based on their distribution in the different provinces. In general, Tetouan province had the most contaminated springs, while those of Chefchaouen were least polluted.

Since fumigants and gasoline hydrocarbons were generally the most prevalent groups, the distribution suggests that VOCs are mainly released from local human activity, such as from agricultural, touristic, and industrial activities. The next section is dedicated to a discussion of the evidence for the potential origins of the predominant compounds.

### 3.2. Frequency of Detection and Potential Origin

The 2,2-DCP, Trans-1,3-DCP, STY, and 1,2-DCP VOCs were the most frequently detected organic volatiles, with frequencies of 38.5%, 23.1%, 23.1%, and 19.2%, respectively. In addition, 1,1-DCE, CHF, NAPH, PCE, and Trans-1,2-DCE shared the same detection frequency of 7.7%. The least detected volatile compounds were DBE, Cis-1,3-DCP, 1,3-DCP, 1,2,4-TMB, 1,3,5-TMB, and CYM, with an equal percentage of 3.8%; i.e., each compound was detected in one sampling site only. These results are in contradiction with earlier findings, wherein chloroform (CHF), toluene, 1,2,4-trimethylbenzene, and perchloroethene (PCE) were the most detected VOCs in non-treated well samples [8].

Interestingly, 75% of the frequently detected VOCs in the investigated samples (2,2-DCP, Trans-1,3-DCP, and 1,2-DCP) belong to the fumigant group. In particular, cis/trans-1,3-DCP is a pesticide used in Morocco largely against nematodes in red-fruit-producing regions (mostly Larache province) [47], constituting 87% of the total import of pesticides used in the agricultural sector [48]. In 2018 alone, the Kingdom of Morocco imported 1350 tons and 1,900,000 L of pesticides containing dichloropropene [49]. Furthermore, agriculture in the Tangier–Tetouan–Al Hoceima region has seen fairly high improvement in the cultivation of crops in 2020–2021, since more than 36,000 hectares of vegetables were planted over 21,000 hectares between September 2020 and February 2021 [50]. Considering the extensive use of fumigants containing 1,2 dichloropropane and 1,3 dichloropropene for tomato production [51], these agricultural activities could be a major factor, if not the only one, causing the occurrence of these chemical compounds in the investigated samples. To emphasize this point, Larache province is well-known for its intensive use of pesticides and irrigation, causing groundwater contamination problems because of its permeable sandy soils [52]. To some extent, this explains the presence of trans-1,3-DCP in S10, S11, and S12, all of which are located in Larache. Another piece of evidence regarding the contamination of water resources by pesticides was provided by Sarti et al. [53], who found trace amounts (still within the permissible limit) of organochlorine in some wells located in the northern region of Morocco. On the other hand, despite the interest in 2,2-DCP, the lack of information about its origins makes it difficult to understand its distribution in several sampling sites.

Styrene, with a range of 2.56–4.09 µg/L, was detected in six of the sampling sites (S7, S8, S10, S11, S13, and S26); most of which are locations frequented by tourists. Concerningly, styrene is a gasoline hydrocarbon that belongs to the BTEX-S group of harmful volatile organics (benzene, toluene, ethylbenzene, three xylene isomers, styrene) that are emitted into the environment (water, soil, air) from several sources [54]. It is a component of cigarette smoke and automobile exhaust, and it may occur naturally at low levels in various types of foods [55]. Given the local touristic and commercial objectives at these zones, styrene may be released due to an abundance of automobile traffic (e.g., dozens of water sellers regularly visiting to supply the urban population with their favorite spring water).

Overall, while most of the volatiles we detected belong to the fumigant group and are principally related to agriculture activities (e.g., tomato, red fruits) that have recently been increasing, automobile exhaust due to, e.g., touristic activities were the main source of styrene in the investigated water samples.

### 3.3. Health Risk Assessment

Cancer risk is the incremental likelihood of developing cancer following lifetime exposure to a specific carcinogen via various exposure routes [16]. According to the EPA [56], the acceptable malignancy risk level ranges between 1 × 10^−6^ and 1 × 10^−4^, while the risk is considered unacceptable if the risk level is higher than 1 × 10^−4^ due to an expected hazard to human health. It should be noted that the data used for the calculation were obtained from the USEPA database and that several VOCs were not included in the assessment due to the unavailability of their reference doses or cancer slope factors [34]. However, Table 2 and Table 3 report the health risk estimates through ingestion and dermal contact of the detected VOCs in the studied areas.

### 3.4. Cancer Risk Assessment

As shown in Table 2, the cancer risk results for the main route (ingestion) revealed that all the samples were suitable for human consumption without any possible health risk. However, despite all studied samples falling within the acceptable range of dermal carcinogenic risk (below the 10^−4^ benchmark level), the risk values for NAPH in S17 (2.1 × 10^−3^), CHF in S18 (2.5 × 10^−4^), and cis and trans-1,3-DCP in S8 (1.61 × 10^−4^) and S19 (1.11 × 10^−4^) posed a relevant skin concern (melanoma).

The blue source or “Aïn Zarka” (S17) in Tetouan is a popular tourist destination mainly known for swimming activities [57]; while its industrial activity is not well-developed, it exhibited a significant quantity of NAPH. This was very likely due to the poor air quality in Tetouan urban areas, which are principally polluted by aerosols from European and African urban areas, forest fires, and the fossil-fuel emissions of commercial ships [58]. Aside from touristic activities, the naphthalene pollution could also have been caused by the extensive wildland fires that broke out in the Tangier–Tetouan–Al Hoceima region in August and September 2020 [59,60]. These findings corroborate those of Proctor et al. [61], who detected naphthalene and other gasoline hydrocarbons in spring waters after a destructive fire. This is also supported by Certini [62], who proved that the bulk soil organic matter from a Quercus ilex forest that had been heated to more than 200 °C became richer in naphthalene and other aromatic hydrocarbons. On the other hand, as the marine and forest environments suggest the natural occurrence of CHF in the terrestrial environment [63,64], the ocean and the Rmilat forest in Tangier might be the practical source of chloroform in S18. In fact, spring waters are not expected to be chlorinated, since they are not provided by public water suppliers [39]. These suppliers generally disinfect drinking water for human health purposes by using chemical disinfectant (mostly chlorine) to eliminate pathogenic organisms [65]. Thus, through drinking water, humans can be exposed to the chloroform that is formed from the reaction of chlorine and organic materials found naturally in raw water supplies [66]. That said, a monitor for one of the sampled springs (S18) claimed [67] that the spring undergoes regular cleaning and coating of its rocks and walls with limewash from time to time, which may contribute significantly to the release of CHF. As chloroform was absent at S19, which is located not far from the Rmilat forest in Tangier, the origin of it at S18 is likely to be release via chlorination.

### 3.5. Non-Cancer Risk Assessment

The computed result for non-carcinogenic risk via ingestion pathway indicated no notable effect (HQ < 1). This result means that the amounts of the studied VOCs were commonly below the level of concern in all samples. However, the observed non-carcinogenic dermal risks (HQ > 1) were mainly attributable to CHF in S18, PCE in S5 and S17, and to NAPH in S17.

CHF, NAPH, and PCE are the three volatiles that pose the greatest dermal risk; as both CHF and NAPH were addressed in Section 3.4 above, we need only discuss the main source of PCE in S5 and S17. S5 is located in an unpopulated area without the presence of the usual activities that produce PCE, such as dry cleaning. Therefore, the high emission ratios from local traffic activities could be the source of this compound [45].

### 3.6. Hierarchical Cluster Analysis of Principal Components (HCPC)

**Figure 5:**
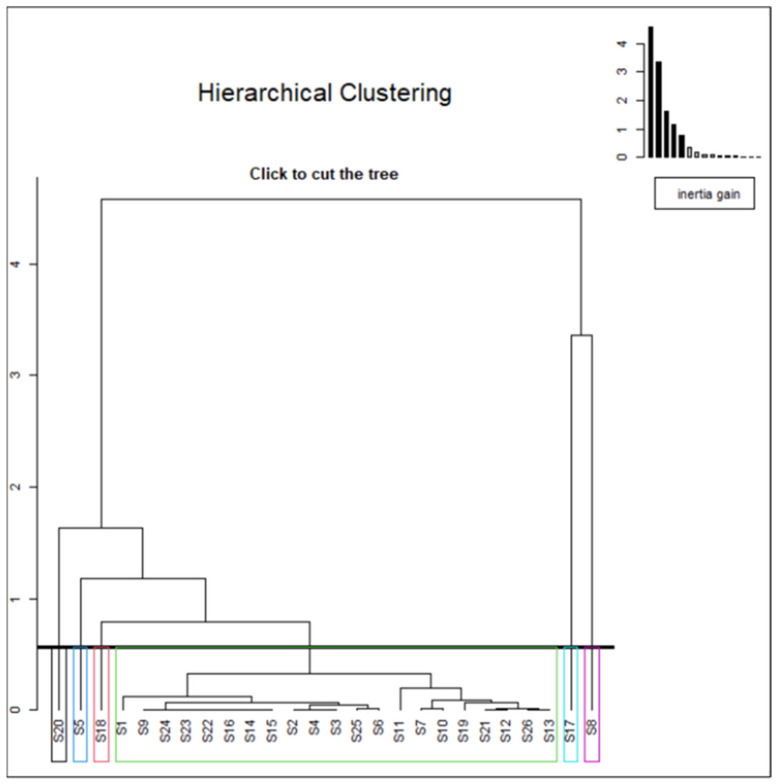
Dendrogram of the two first principal components (PC1 and PC2) of the studied sampling sites.

The hierarchical cluster analysis of principal components (HCPC) was performed on VOC data to classify the sampling sites according to their similarities and to understand their variability. To minimize the total within-cluster variability, Ward’s criterion was selected to build the hierarchical tree [68]. The dendrogram (Figure 5) was plotted on the two first principal axes, accounting for 55.6% of the total variation with 32% and 26.5% for PC1 and PC2, respectively, and clustered the analyzed samples into six main groups (from left to right: 1 to 6).

The first cluster includes a single sample (S20), which is distinguished by the presence of 1,1-DCE and 1,2,4-TMB in amounts that are much higher than the overall average across all the clusters. In cluster 2, the spring water (S5) has the highest CYM and PCE amounts, more than the overall average, but is principally distinguished by the occurrence of CYM. In cluster 3 (S18), the chloroform amount is the most significantly associated variable. The fourth cluster is sorted into twenty-one sampling sites from distinct provinces, which, in turn, are divided into two main sub-classes. Generally, compared to all other sites, this group is mainly characterized by the lower amount of NAPH, CYM, 1,3,5-TMB, 1,2,4-TMB, 1,3-DCP, Cis-1,3-DCP, DBE, PCE, and trans-1,2 DCE compared to the overall mean.

Clusters 5 and 6 are comprised of one site only: S17 and S8, respectively. The concentration values of trans-1,2-DCE and 1,2-DCP are higher in both clusters compared to the average of all the clusters. However, versus the overall average, what discriminates these last groups was the high amount of NAPH, 1,3-DCP, and PCE in group 5 and of 1,3,5-TMB, cis-1,3-DCP, DBE, trans-1,3-DCP, and STY in group 6. Considering that the cannabis monoculture system involves such intensive use of pesticides that the runoff infiltrates and pollutes the water system [69], the occurrence of three fumigants (cis-1,3-DCP, DBE, trans-1,3-DCP) in S8 (cluster 6) can be principally attributed to the use of pesticides in this rural area (located in Tetouan province). Since the 1980s, the city in this area has extensively developed its cannabis cultivation, contributing 7% of total Moroccan production [70]; it currently contributes about 4% of total raw cannabis production in Morocco [71]

The primary use of naphthalene is in the production of phthalic anhydride, carbamate insecticides, surface active agents and resins, miscellaneous organic chemicals, and as a dye intermediate, a synthetic tanning agent, and a moth repellent. Fossil fuels and burning tobacco or wood are also sources of emission [72,73], as well as vehicular release in urban areas [74]. PCE is also released from traffic-related sources [45] and is used as a dry-cleaning agent, chemical intermediate, and degreasing agent for metals [75]. Altogether, the origins of NAPH, 1,3-DCP, and PCE at S17 could be related to agricultural activities, atmospheric pollution, and some chemical intermediates.

Overall, based on the HCPC analysis, twenty-one spring water samples showed some similarities, but the water quality at S5, S8, S17, S18, and S20 differed from one site to another due to differences in local anthropogenic sources of contamination.

## 4. Conclusions

This study aimed to uncover evidence of possible sources of VOCs found at the studied springs. The potential health risk was evaluated through two main pathways: ingestion and dermal. One of the most significant findings to emerge from this investigation was that seven of the investigated samples were VOC-free. The most detected VOCs belonged to the fumigants group and are mainly leached from soil that has been contaminated by the extensive use of pesticides in agricultural activities, e.g., cannabis and red fruit cultivations. While health risk assessment revealed all studied water samples to be harmless in terms of the two studied pathways, S17 (naphthalene), S18 (chloroform), S8 and S19 (cis and trans-dichlropropene) showed a carcinogenic dermal risk. For non-carcinogenic risk, water from three sampling sites might have an impact on skin. 

Overall, chemical chlorination, forest fires, and local traffic emissions were the main origins of the harmful compounds that we identified. Interestingly, the twenty-six sampling sites were successfully classified into six well-discriminated clusters via HCPC analysis. The heatmap also proved to be a useful tool to distinguish between the provinces based on their degree of pollution for each of the five VOCs groups.

In conclusion, insofar as water is fundamental to human life, it is mandatory to preserve it and ensure its safety. Therefore, to establish a more accurate appraisal of the human health risks, we highly recommend further detailed assessment of the differences in VOC exposure for different seasons. In addition, authorities and decision-makers in the Tangier–Tetouan–Al Hoceima region should give considerable attention to the quality of these uncontrolled spring waters by limiting the different sources of their pollution that threaten the health of exposed inhabitants.

## Figures and Tables

**Table 1:** Concentrations of 15 VOCs in water samples, their frequency of occurrence, total amounts of each sampling site, classification [3], abbreviation, WHO drinking water quality standards [35], and IARC classification of the targeted VOCs.

	Organic Synthesis Compounds	Trihalomethanes	Fumigant						Gasoline Hydrocarbon			Solvents				Total VOCs
Samples	1,1-DCE	CHF	2,2-DCP	1,2-DCP	DBE	Cis-1,3-DCP	Trans-1,3-DCP	1,3-DCP	NAPH	STY	1,2,4-TMB	PCE	Trans-1,2-DCE	1,3,5-TMB	CYM	
Sample n1	<MDL	2.79 ± 0.36	1.70 ± 0.2	<MDL	<MDL	<MDL	<MDL	<MQL	<MQL	<MQL	<MDL	<MDL	<MQL	<MDL	<MDL	4.49
Sample n2	<MDL	<MDL	1.44 ± 0.01	<MDL	<MDL	<MDL	<MDL	<MDL	<MDL	<MDL	<MDL	<MDL	<MQL	<MDL	<MDL	1.44
Sample n3	<MDL	<MDL	1.26 ± 0.14	<MDL	<MDL	<MDL	<MDL	<MQL	<MQL	<MDL	<MDL	<MDL	<MDL	<MDL	<MDL	1.26
Sample n4	<MDL	<MDL	1.14 ± 0.08	<MDL	<MDL	<MDL	<MDL	<MQL	<MDL	<MDL	<MDL	<MQL	<MQL	<MQL	<MDL	1.14
Sample n5	<MDL	<MDL	1.42 ± 0.07	<MDL	<MDL	<MDL	<MDL	<MQL	<MQL	<MDL	<MDL	1.40 ± 0.17	<MDL	<MDL	0.27 ± 0.01	3.09
Sample n6	<MDL	<MDL	1.22 ± 0.17	0.61 ± 0.14	<MDL	<MDL	<MDL	<MQL	<MDL	<MQL	<MDL	<MQL	<MQL	<MDL	<MDL	1.83
Sample n7	<MDL	<MDL	1.58 ± 0.15	<MDL	<MDL	<MDL	<MDL	<MQL	<MQL	4.09 ± 0.35	<MDL	<MQL	<MQL	<MDL	<MDL	5.67
Sample n8	<MDL	<MDL	1.52 ± 0.04	0.72 ± 0.05	1.36 ± 0.02	0.19 ± 0.00	1.12 ± 0.13	<MQL	<MQL	3.83 ± 0.58	<MDL	<MQL	1.29 ± 0.1	0.41 ± 0.04	<MDL	10.44
Sample n9	<MDL	<MDL	<MQL	<MDL	<MDL	<MDL	<MDL	<MQL	<MDL	<MDL	<MDL	<MQL	<MQL	<MDL	<MDL	-----
Sample n10	<MDL	<MDL	1.33 ± 0.04	<MDL	<MDL	<MDL	0.72 ± 0.1	<MDL	<MDL	3.13 ± 0.09	<MDL	<MDL	<MQL	<MDL	<MDL	5.18
Sample n11	2.03 ± 0.12	<MDL	<MQL	<MDL	<MDL	<MDL	0.69 ± 0.00	<MDL	<MDL	2.77 ± 0.15	<MDL	<MDL	<MQL	<MDL	<MDL	5.49
Sample n12	<MDL	<MDL	<MQL	<MDL	<MDL	<MDL	0.79 ± 0.07	<MDL	<MDL	<MQL	<MDL	<MDL	<MQL	<MDL	<MDL	0.79
Sample n13	<MQL	<MDL	<MQL	<MDL	<MDL	<MDL	<MDL	<MDL	<MDL	3.6 ± 0.29	<MDL	<MDL	<MQL	<MDL	<MDL	3.6
Sample n14	<MDL	<MDL	<MQL	<MDL	<MDL	<MDL	<MDL	<MDL	<MDL	<MDL	<MDL	<MDL	<MQL	<MDL	<MDL	-----
Sample n15	<MDL	<MDL	<MDL	<MDL	<MDL	<MDL	<MDL	<MDL	<MDL	<MDL	<MDL	<MDL	<MQL	<MDL	<MDL	-----
Sample n16	<MDL	<MDL	<MQL	<MDL	<MDL	<MDL	<MDL	<MQL	<MDL	<MDL	<MDL	<MQL	<MQL	<MDL	<MDL	-----
Sample n17	<MDL	<MDL	1.9 ± 0.28	0.80 ± 0.04	<MDL	<MDL	<MDL	1.24 ± 0.01	2.26 ± 0.38	<MQL	<MDL	2.86 ± 0.06	1.46 ± 0.04	<MQL	<MDL	10.52
Sample n18	<MDL	7.6±0.00	<MQL	<MDL	<MDL	<MDL	<MDL	<MQL	<MDL	<MDL	<MDL	<MQL	<MQL	<MDL	<MDL	7.6
Sample n19	<MDL	<MDL	<MQL	<MDL	<MDL	<MDL	0.91 ± 0.07	<MQL	<MDL	<MDL	<MDL	<MQL	<MQL	<MDL	<MDL	1.68
Sample n20	2.76±0.16	<MDL	<MQL	<MDL	<MDL	<MDL	<MDL	<MDL	<MDL	<MQL	0.27 ± 0.00	<MDL	<MQL	<MDL	<MDL	3.03
Sample n21	<MDL	<MDL	<MQL	<MDL	<MDL	<MDL	0.66 ± 0.03	<MDL	<MDL	<MDL	<MDL	<MDL	<MQL	<MDL	<MDL	0.66
Sample n22	<MDL	<MDL	<MQL	<MDL	<MDL	<MDL	<MDL	<MDL	<MDL	<MQL	<MDL	<MDL	<MQL	<MDL	<MDL	-----
Sample n23	<MQL	<MDL	<MQL	<MDL	<MDL	<MDL	<MDL	<MDL	<MDL	<MQL	<MDL	<MDL	<MQL	<MDL	<MDL	-----
Sample n24	<MQL	<MDL	<MQL	<MDL	<MDL	<MDL	<MDL	<MDL	<MDL	<MQL	<MDL	<MDL	<MQL	<MDL	<MDL	-----
Sample n25	<MDL	<MDL	<MQL	0.48 ± 0.02	<MDL	<MDL	<MDL	<MQL	<MDL	<MQL	<MDL	<MDL	<MDL	<MDL	<MDL	0.48
Sample n26	<MQL	<MDL	<MQL	<MDL	<MDL	<MDL	<MDL	<MDL	<MDL	2.56 ± 0.14	<MDL	<MDL	<MDL	<MDL	<MDL	2.56
Min	<MDL	<MDL	<MDL	<MDL	<MDL	<MDL	<MDL	<MDL	<MDL	<MDL	<MDL	<MDL	<MDL	<MDL	<MDL	
Max	2.76	7.6	1.9	0.8	1.36	0.19	1.12	1.24	2.26	4.09	0.27	2.86	1.46	0.41	0.27	
FOD (%)	7.69	7.69	38.46	19.23	3.85	3.85	23.08	3.85	7.69	23.08	3.85	7.69	7.69	3.85	3.85	
WHO (µg/L)	30	200	-----	40	-----	-----	-----	-----	-----	20	-----	40	50 (cis and trans)	-----	-----	
IARC classification	-----	2B	-----	1	-----	2B	2B	-----	2B	2A	-----	2A	-----	-----	-----	

1,1-DCE: 1,1-dichloroethene, CHF: chloroform, 2,2-DCP: 2,2-dichloropropane, 1,2- DCP: 1,2-dichloropropane, DBE: dibromomethane, Cis-1,3-DCP: 1,3-dichloropropene Z, Trans-1,3-DCP: 1,3-dichloropropene E, 1,3-DCP: 1,3-dichloropropane, NAPH: naphthalene, STY: styrene, 1,2,4-TMB: 1,2,4-trimethylbenzene, PCE: tetrachloroethylene, Trans-1,2-DCE: 1,2-dichloroethelene E, 1,3,5-TMB: 1,3,5-trimethylbenzene, CYM: cymene, FOD: frequency of detection, WHO: World Health Organizartion Standard, IARC classification: International Agency for Research on Cancer calassification.

**Table 2:** Portability of carcinogenic risks of VOCs in the studied areas.

Carcinogenic Risk Assessment						
Samples	Pathway	CHF	1,2-DCP	Cis Trans-1,3-DCP	PCE	NAPH
Sample n1	Ingestion	2.47 × 10^−6^				
	Dermal	9.21 × 10^−5^				
Sample n5	Ingestion				8.42 × 10^−8^	
	Dermal				2.09 × 10^−5^	
Sample n6	Ingestion		6.41 × 10^−7^			
	Dermal		2.62 × 10^−8^			
Sample n8	Ingestion		7.59 × 10^−7^	3.7 × 10^−6^		
	Dermal		3.10 × 10^−8^	1.61 × 10^−4^		
Sample n10	Ingestion			2.6 × 10^−6^		
	Dermal			8.8 × 10^−5^		
Sample n11	Ingestion			2 × 10^−6^		
	Dermal			8.46 × 10^−5^		
Sample n12	Ingestion			2.3 × 10^−6^		
	Dermal			9.71 × 10^−5^		
Sample n17	Ingestion		8.51 × 10^−7^		1.72 × 10^−7^	7.75 × 10^−6^
	Dermal		3.48 × 10^−8^		4.25 × 10^−8^	2.10 × 10^−3^
Sample n18	Ingestion	6.72 × 10^−6^				
	Dermal	2.50 × 10^−4^				
Sample n19	Ingestion			2.6 × 10^−6^		
	Dermal		3.33 × 10^−8^	1.11 × 10^−4^		
Sample n20	Ingestion					
	Dermal					
Sample n21	Ingestion			1.9 × 10^−6^		
	Dermal			8.17 × 10^−5^		
Sample n25	Ingestion		5.03 × 10^−7^			
	Dermal		2.06 × 10^−8^			

**Table 3:** Probability of non-carcinogenic risks of VOCs in the studied areas.

Non-Carcinogenic Risk Assessment												
Samples	Pathway	1,1-DCE	Trans-1,2-DCE	CHF	1,2-DCP	Cis Trans 1,3-DCP	1,3-DCP	PCE	STY	1,2,4-TMB	1,3,5-TMB	NAPH
Sample n1	Ingestion			7.98 × 10^−3^								
	Dermal			6.93 × 10^−1^								
Sample n5	Ingestion							6.68 × 10^−3^				
	Dermal							3.86 × 10^+1^				
Sample n6	Ingestion											
	Dermal				4.14 × 10^−2^							
Sample n7	Ingestion								5.84 × 10^−4^			
	Dermal								2.53 × 10^−1^			
Sample n8	Ingestion		1.84 × 10^−3^		5.13 × 10^−4^	1.2 × 10^−3^			5.48 × 10^−4^		1.18 × 10^−3^	
	Dermal		2.24 × 10^−1^		4.89 × 10^−2^	1.3 × 10^−1^			2.37 × 10^−1^		9.38 × 10^−1^	
Sample n10	Ingestion					6.8 × 10^−4^			4.48 × 10^−4^			
	Dermal					6.8 × 10^−2^			1.94 × 10^−1^			
Sample n11	Ingestion	1.2 × 10^−3^				6.6 × 10^−4^			3.96 × 10^−4^			
	Dermal	8.69 × 10^−2^				6.6 × 10^−2^			1.71 × 10^−1^			
Sample n12	Ingestion					7.5 × 10^−4^						
	Dermal					7.6 × 10^−2^						
Sample n13	Ingestion								5.14 × 10^−4^			
	Dermal								2.22 × 10^−1^			
Sample n17	Ingestion		2.09 × 10^−3^		5.75 × 10^−4^		1.8 × 10^−3^	1.36 × 10^−2^				3.23 × 10^−3^
	Dermal		2.5 × 10^−1^		5.49 × 10^−2^		1.68 × 10^−1^	7.88 × 10^+1^				2.04 × 10^+1^
Sample n18	Ingestion			2.17 × 10^−2^								
	Dermal			1.88 × 10^+1^								
Sample n19	Ingestion					8.7 × 10^−4^						
	Dermal				5.26 × 10^−2^	8.7 × 10^−2^						
Sample n20	Ingestion	1.6 × 10^−3^								7.59 × 10^−4^		
	Dermal	1.18 × 10^−1^								8.31 × 10^−1^		
Sample n21	Ingestion					6.3 × 10^−4^						
	Dermal					6.4 × 10^−2^						
Sample n25	Ingestion				3.40 × 10^−4^							
	Dermal				3.25 × 10^−2^							
Sample n26	Ingestion								3.66 × 10^−4^			
	Dermal								1.58 × 10^−1^			

## Data Availability

Not applicable.

## Supplementary Materials

The following supporting information can be downloaded at: https://www.mdpi.com/article/10.3390/metabo12121213/s1, Table S1: Additional information on the studied springs according to High Commission for Planning of Morocco (2019), Table S2: Data of qualitative ion, quantitative ion, R^2^, retention times, MDL, MQL of 15 detected VOCs, Table S3: Parameters and input assumptions for exposure assessment of Volatile Organic Compounds through ingestion and dermal pathways, and Table S4: Slope factor, oral reference dose, and dermal permeability coefficient of 15 detected VOCs.
