# Supplementary material for: Preliminary Assessment of Occurrence, Potential Origin, and Human Health Risk of Volatile Organic Compounds in Uncontrolled Springs, North Morocco
Source: Metabolites. 2022 Dec 2;12(12):1213. doi: 10.3390/metabo12121213 (PMC9788112; doi:10.3390/metabo12121213)
Supplement: Supplementary file 1 [file metabolites-12-01213-s001.zip › metabolites-2034777-supplementary.pdf]

Supplementary Material

Table S1. Additional information on the studied springs according to High Commission for Planning of Morocco (2019) [30].

	Point	Latitude	Longitude	Altitude: (m.)	Province/Prefecture	Rural commune	Douar (Countryside)	Population	Households	Households connected to public water supply (%)	Wastewater disposal Methods (%)			Methods of Domestic Waste Disposal (%)			Type of Springs*	Consumers *
											Public Sewerage System	Septic tank	Other	Municipal Trash Cans	Municipality truck / Private truck	others		
S1	Ben karrich	35.52126763	- 5.43578466	71.54	Tetouan	Urban	Dar Bni Karrich (Center)	X	X	X	X	X	X	X	X	X	BCW	Pass by the Spring
S2	Ain Abdouss	35.31009636	-5.35177773	575.46	Tetouan	Urban		X	X	X	X	X	X	X	X	X	RS	Pass by the Spring
S3	Ain El Rami	35.12534019	-5.28815328	385.62	Chefchaouen	Bab Taza	Ain El Rami	138	34	0	0	97.1	2.9	8.8	0	91.2	SB	Vendors, and fans from Chefchaouen
S4	Ain Maakacha	35.0422924	-5.18209398	750.06	Chefchaouen	Bab Taza	Maakacha	271	48	6.3	0	97.9	2.1	0	0	100	SB	Only the inhabitants
S5	Ain Bab Elkaren	35.00657894	-5.20258306	1248.6	Chefchaouen	Bab Taza	Bouzthate	2,516	455	0.2	0.2	93.2	6.6	0	0.2	99.8	RS	An immense number of Spring's fans
S6	Ain Lefhama	34.70695933	-5.40216789	295.73	Ouazzane	Sidi Redouane	Ouled Abdellah	544	141	0	0	94.3	5.7	0	0	100	BCW	Inhabitants and Spring's fans from Zouakiyene
S7	Ain Sedine	34.63999339	-5.54160922	92.5	Sidi Kacem	Ain Defali	Jeramena	449	94	2.1	0	27.7	72.3	0	0	100	BCF	Inhabitants, Vendors and fans from Jorf El Melha (Mun.), Had Kourt (Mun.), Sidi Kacem, Sidi Slimane, and Mechra Bel Ksiri.
S8	Ain Bghaghza	35.43296292	-5.67284271	604.99	Tetouan	Bghaghza	Esqaryech	534	108	0	0.9	93.5	5.6	0.9	0	99.1	RS	An immense number of vendors and fans from Tangier
S9	Franco Alhar	35.552814	-5.88883896	6.31	Tangier-Asilah	El Manzala	El Har	208	43	0	0	97.7	2.3	0	0	100	RS	Inhabitants and Spring's fans form Tangier
S10	Saniat ElKifan	35.30220499	-5.84639943	279.66	Larache	Beni Garfett	Al Kifane	1,256	277	0	0	31	69	0	0	100	RS	Only the inhabitants
S11	Ain Olika Bouhani	35.28906826	-5.82958432	327.81	Larache	Beni Garfett	Bouhani	899	183	0.5	1.6	85.8	12.6	0	0	100	BCW	Spring's fans form Tangier and Larache
S12	Ain Khamassa	35.21394433	-6.01861211	54.54	Larache	Sahel	Lbelat	512	116	5.2	0.9	12.9	86.2	0	0	100	BCW	Vendors, Spring's fans form Tangier, Larache, and neighborhood
S13	Ain Ozid	35.42066794	-5.60676066	805.42	Tetouan	Bghaghza	Zaouia Foukia	285	62	0	0	1.6	98.4	0	0	100	RS	Inhabitants, Vendors, Spring's fans form Tangier
S14	Ain Ahjij	35.74210663	-5.63630559	166.72	Fahs Anjra	Melloussa	Ain Said	497	116	36.2	0	92.2	7.8	0	0	100	BCF	Only the inhabitants
S15	Ain Rmel	35.77283247	-5.56901504	213.58	Fahs Anjra	Ksar Sghir	Ain Rmel	989	208	4.8	36.1	0.5	62	1.4	1.9	94.2	RS	Inhabitants, some vendors and fans from Tangier
S16	Ain Sonbol	35.61168511	-5.38419288	246.36	Tetouan	El Malaliyine	Al Maeadil	219	38	0	0	97.4	2.6	0	0	100	RS	Inhabitants, some vendors and fans from Tetouan and M'diq
S17	Ain Zerka	35.52051606	-5.34300645	141.61	Tetouan	Zaitoune	Yarghit	847	177	2.8	74.6	14.7	10.7	58.2	1.7	40.1	BCW	Some inhabitants, Spring's fans and Visitors of Zerka
S18	Ain Lala Yeno	35.78959706	-5.84673151	139.03	Tangier-Asilah	Urban		X	X	X	X	X	X	X	X	X	SB	Spring's fans from Tangier
S19	Ain Lehri	35.77167279	-5.91222876	163.03	Tangier-Asilah	Urban		X	X	X	X	X	X	X	X	X	SB	Only a few inhabitants
S20	Ain Bahri	35.41193674	-6.04881068	96.69	Tangier-Asilah	Sahel Chamali	Tendafel	563	135	0	0	39.3	60.7	0	0	100	RS	Inhabitants and some spring's fans
S21	Cheraka	35.68162234	-5.89075399	86.6	Tangier-Asilah	Ahejarr Ennehal	Chraka	2,451	543	11.4	1.1	77	21.9	1.1	0	98.9	BCF	Some inhabitants and few fans from Tangier

S22	Ain Rondi_	35.57763626	-5.74918533	165.61	Tangier-Asilah	Dar Chaoui	EL Ramla	844	164	0	0	93.3	6.7	0	0	100	SB	Only a few inhabitants
S23	Ain khandek Snane	35.54151471	-5.76379724	178.59	Tangier-Asilah	Dar Chaoui	Khandek Snane	X	X	X	X	X	X	X	X	X	BCF	Only the inhabitants
S24	Ain Sedrauoia	35.32325054	-5.53115623	1061.28	Larache	X	X	F	X	X	X	X	X	X	X	X	SB	Spring's fans form Larache region and pass by
S25	Ain Oued Setah	35.27650635	-5.53204554	757.8	Larache	X	X	F	X	X	X	X	X	X	X	X	BCF	Spring's fans form Larache region and vendors form Larache and Tetouan
S26	Ain Bouhachem	35.30116359	-5.50989342	1091.74	Larache	X	X	F	X	X	X	X	X	X	X	X	BCF	Spring's fans form Larache region and vendors form Larache and Tetouan

BCF: Box constructed for collection; springs are supplied with faucet, BCW: Box constructed for collection; springs are supplied without faucet,RS: Running Springwater, SB: Spring bubbled up from the earth. *: Authors' investigations, X: unknown, F: located in the forest

Table S2. Data of qualitative ion, quantitative ion, R^2, retention times, MDL, MQL of 15 detected VOCs.

HS-SPME-GC-MS method						
Compounds	Qualitative ion	Quantitative ion	R^2	Retention time (min)	MDL (ppb)	MQL (ppb)
1,1-dichloroethylene	61	96, 98	0.9987	3.44	0.55	1.83
trans-1,2 dichloroethylene	61	96, 98	0.9976	4.65	0.74	2.45
2,2-dichloropropane	77	41, 79	0.9871	6.54	0.32	1.05
Chloroform	83	85	0.996	7.16	0.25	0.82
1,2-dichloropropane	63	62,76	1	9.5	0.1	0.32
Dibromomethane	174	93	0.998	9.84	0.21	0.70
cis-1,3-dichloropropene	75	39, 110	1	10.98	0.01	0.04
trans-1,3-dichloropropene	75	39, 110	0.999	12.04	0.11	0.36
Tetrachloroethene	166	131,94	0.9982	12.64	0.51	1.69
1,3-dichloropropane	76	41,78	0.999	12.64	0.37	1.24
Styrene	104	104, 78	0.9963	15.28	0.72	2.39
1,2,4-trimethylbenzene	105	120	0.9974	17.37	0.61	2.02
1,3,5-trimethylbenzene	105	120	0.9978	18.10	0.56	1.86
Cymene	119	134	0.9971	18.73	0.64	2.14
Naphthalene	128	102	0.9988	23.12	0.41	1.35

Table S3. Parameters and input assumptions for exposure assessment of Volatile Organic Compounds through ingestion and dermal pathways [32,33]

Parameter	Unit	Values	
		Ingestion	Dermal adsorption
Concentrations of Volatile organic compounds (Cw)	μg/L	–	–
Ingestion Rate (Daily average intake) (IR)	L/day	2	–
Exposure frequency (EF)	Day/years	365	350
Exposure duration (ED)	Year	70	30
Body weight (BW)	Kg	70	70
Averaging time (AT)	Days	25550	10950 (ED×365)
Skin-surface area (SA)	Cm2	–	18000
Event time (ET)	hr/event	–	0.58 hr/event (35 minutes/event)
Event frequency (EF):	Events/day		1.0
Exposure frequency (EF):	Days/year		350.0
Exposure duration	Years	–	30
Skin thickness (assumed to be 10 um) (ISC)	cm		1.00E-03
Absorbed cancer slope factor (SFABS)	(mg/kg-day)-1		Chemical specific
Oral Cancer Slope Factor (SFo)	(mg/kg-day)		Chemical specific
Fraction of contaminant absorbed in gastrointestinal tract in the critical toxicity study (ABSGI)	(dimensionless)		Chemical specific
Absorbed reference dose (RfDABS)	mg/kg-day		Chemical specific
Reference dose oral (RfDO)	mg/kg-day		Chemical specific
Absorption Fraction (AF)	(unitless)		Chemical specific
Dermal permeability coefficient (Kp)	(cm/hr)		Chemical specific

Averaging time (days) for carcinogenic effects, AT=70 years (25,550 days) and for noncarcinogenic effects, AT=ED (in days). While, Exposure duration (years) for carcinogenic effects, ED = 30 years and for noncarcinogenic effects.

Table S4. Slope factor, oral reference dose, and dermal permeability coefficient of 15 detected VOCs [34]

Compounds	Slope factor	Oral reference dose	Dermal permeability coefficient (cm/hr)
1,1-dichloroethylene		$5x10^{-2}$	$6.8x10^{-3}$
trans-1,2 dichloroethylene		$2x10^{-2}$	$1.1x10^{-2}$
2,2-dichloropropane			
Chloroform	$3.1x10^{-2}$	$1x10^{-2}$	$6.83x10^{-3}$
1,2-dichloropropane	$3.7x10^{-2}$	$4x10^{-2}$	$7.8x10^{-3}$
Dibromomethane			
cis-1,3-dichloropropene	$1x10^{-1}$	$3x10^{-2}$	$8.34x10^{-3}$
trans-1,3-dichloropropene	$1x10^{-1}$	$3x10^{-2}$	$8.34x10^{-3}$
Tetrachloroethene	$2.1x10^{-3}$	$6x10^{-3}$	$3.3x10^{-2}$
1,3-dichloropropane		$2x10^{-2}$	$7.76x10^{-3}$
Styrene		$2x10^{-1}$	$3.7x10^{-2}$
1,2,4-trimethylbenzene		$1x10^{-2}$	$8.57x10^{-2}$
1,3,5-trimethylbenzene		$1x10^{-2}$	$6.21x10^{-2}$
Cymene			
Naphthalene	$1.2x10^{-1}$	$2x10^{-2}$	$4.7x10^{-2}$
